# Convolution Neural Network with Laser-Induced Breakdown Spectroscopy as a Monitoring Tool for Laser Cleaning Process

**DOI:** 10.3390/s23010083

**Published:** 2022-12-22

**Authors:** Soojin Choi, Changkyoo Park

**Affiliations:** 1 Department of Laser and Electron Beam Technologies, Korea Institute of Machinery and Materials, Daejeon 34103, Republic of Korea; 2 Department of Materials Science and Engineering, Seoul National University of Science and Technology, Seoul 01811, Republic of Korea

**Keywords:** laser cleaning, paint removal, monitoring, laser-induced breakdown spectroscopy, convolution neural network

## Abstract

In this study, eight different painted stainless steel 304L specimens were laser-cleaned using different process parameters, such as laser power, scan speed, and the number of repetitions. Laser-induced breakdown spectroscopy (LIBS) was adopted as the monitoring tool for laser cleaning. Identification of LIBS spectra with similar chemical compositions is challenging. A convolutional neural network (CNN)-based deep learning method was developed for accurate and rapid analysis of LIBS spectra. By applying the LIBS-coupled CNN method, the classification CNN model accuracy of laser-cleaned specimens was 94.55%. Moreover, the LIBS spectrum analysis time was 0.09 s. The results verified the possibility of using the LIBS-coupled CNN method as an in-line tool for the laser cleaning process.

## 1. Introduction

Laser cleaning is the technique to remove contaminants from surfaces by laser ablation, which occurs when a high-energy laser pulse irradiates the sample surface [1]. Laser ablation removes contaminants via the materials’ evaporation and volatilization. Laser cleaning has been widely used in art restorations [2,3], paint removal [4], and maintenance of metal alloys [5]. Monitoring techniques have been applied for overcleaning prevention and residual contaminant detection for laser cleaning. Acoustic signal [4] and FE-SEM and EPMA [6] were used to monitor natural marine microbiofoulings and paint removal on metal surface, respectively. Micro-CT and micro-XRF [7] were adopted to examine the laser cleaning level of black crusts on limestone monuments. However, those techniques may not be suitable as an in-line monitoring for the laser cleaning process. This is because the high-power laser cleaning process produces extremely large noise. In addition, the above-mentioned techniques (except acoustic signal) require thorough pretreatments to obtain accurate analysis results.

Laser-induced breakdown spectroscopy (LIBS) is an effective technique as an in-line monitoring for the laser cleaning process by investigating the elemental composition using laser-induced plasma. Rapid spectral data analysis allows LIBS to be applied for an in-line monitoring. However, identifying different types of samples with similar elemental compositions using LIBS analysis is challenging. Therefore, machine learning and deep learning methods are being adopted to improve the accuracy and speed of LIBS analyses. Sirven et al. [8] adopted principal component analysis (PCA), soft independent modeling of class analogy (SIMCA), and partial least-squares discriminant analysis (PLS-DA) for rock classification. Yelameli et al. [9] used a support vector machine (SVM) to distinguish ten different rock samples. Li et al. [10] applied k-nearest neighbors (kNN) and an SVM to discriminate soft tissues. 

In recent years, the LIBS spectra analysis using convolution neural networks (CNN) has been attempted [11,12,13,14,15,16,17]. Chen et al. [11] adopted a CNN to classify rock samples. The results of the CNN were better than those of other methods, such as kNN, PCA-kNN, and SVM. Feng et al. [12] used the SVM, logistic regression (LR), and CNN to investigate rice leaf diseases. Cao et al. [13] and Castorena et al. [14] performed quantitative analysis of ChemCam (an instrument on the Mars rover Curiosity) spectral data using a CNN. Zhao et al. [15] employed a CNN to predict the brand of iron ore. Xing et al. [16] quantitatively analyzed LIBS data to determine lithium in brine samples. Huang et al. [17] adopted machine learning and CNN methods to identify adulterated milk powders. The use of LIBS spectra analysis with CNN is increasing; however, to the best of the authors’ knowledge, it has not been applied to evaluate the paint removal level using a high-power laser.

We prepared eight different laser paint-cleaned stainless steel 304L (SS304L) with different process parameters such as laser power, scan speed, and the number of repetitions. The LIBS spectra were acquired at the surface of the laser-cleaned specimens. The LIBS analysis was coupled with a CNN to improve the evaluation accuracy and measurement speed of paint removal level. The LIBS analysis with CNN classified eight different specimens with an accuracy of 94.55%, and the analysis time was only 0.09 s per spectrum. These results overcome the limitations of LIBS as a tool for an in-line monitoring. This study verified the feasibility of the LIBS-coupled CNN technique as an in-line monitoring tool for the laser cleaning process. 

## 2. Materials and Methods

### 2.1. Materials and Experimental Setup for Laser Cleaning

Commercial stainless steel 304L (SS304L, POSCO) was painted with red epoxy paint (EH2350, KCC). A 1.2 kW Q-switched Nd:YAG (Rigel i1200, PowerLase) was used for the laser cleaning process. A pulse duration and a wavelength were 89 ns and 1064 nm, respectively. The specimens were irradiated using a laser power of 400 or 500 W with a pulse frequency of 8 kHz. A F-theta lens with a focal length of 163 mm was used to produce a laser beam size of 2.1 mm. A two-dimensional galvanometer scanner (SUPERSCAN IIE-30, RayLase) was used to scan the laser beam. Eight different laser-cleaned (LC) specimens were produced using the laser process parameters listed in Table 1. The laser energy input was calculated using Equation (1). An area of 40 × 40 mm^2^ was cleaned using a laser. All the experiments were performed in air at atmospheric pressure and room temperature.

(1) $$Laser\;energy\;input\;\left(J/\mathrm{mm}\right)=\frac{Laser\;power\;\left(W\right)}{Scan\;speed\;\left(\frac{\mathrm{mm}}{s}\right)\;}\times Number\;of\;repetitions$$

### 2.2. Laser-Induced Breakdown Spectroscopy

LIBS with a Q-switched Nd: YAG laser (VIRON, Quantel Laser by LUMIBIRD, Bozeman, MT, USA) was used to investigate the paint removal level. A pulse duration and a wavelength were 7 ns and 1064 nm, respectively. A laser with a pulse energy of 5 mJ, a pulse frequency of 1 Hz, and a beam size of 50 μm was irradiated on the specimens through a plano-convex lens with a focal length of 100 mm. A spectrometer (IsoPlane 320, Teledyne Prinston Instruments, Trenton, NJ, USA) coupled with an intensified CCD (PI MAX 4, Prinston Instruments) was employed to acquire the laser-induced plasma. The light emitted from the plasma passed through a fiber optic cable with a core size of 600 μm to the spectrometer. A spectral range was 370–630 nm, and a plasma signal was dispersed by a 150 g/mm grating. A delay time of 1 µs and a gate width of 5 µs were applied. The specimens were placed on an X- and Y-axis motorized stage such that the sample location was moved after each laser pulse, and the step size was 1 mm. All the experiments were performed in air at atmospheric pressure and room temperature. Seventy-five of LIBS spectra and one hundred of LIBS spectra were acquired from base metal (BM) and LC specimens, respectively. Each LIBS spectrum was obtained by a single-laser-shot. The information of peaks (e.g., wavelength and intensity) in the LIBS spectrum provides information of the chemical composition of each specimen.

### 2.3. CNN Classification Model

**Figure 1:**
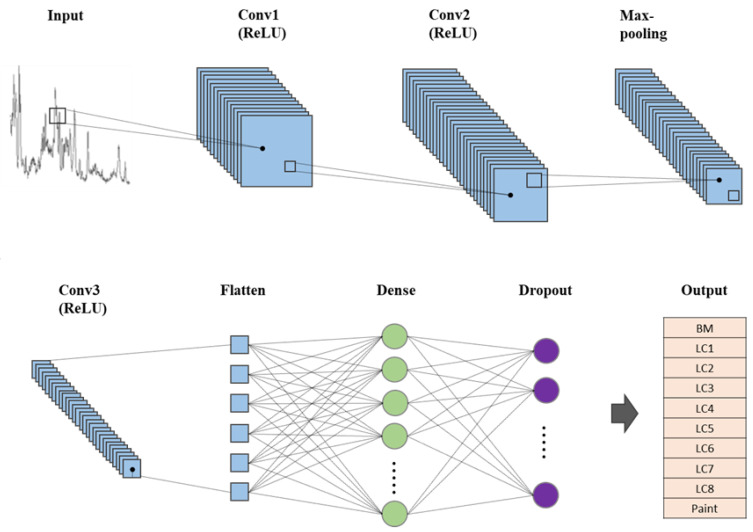
The structure of CNN model.

CNN is a deep learning method widely used to classify images. Figure 1 shows the structure of the CNN model used in this study. The LIBS spectra were used as the input data. The convolution layers were the major layers in the CNN structure used to extract the features of the input data. The input layer followed two convolution layers (Conv1 and Conv2, 3 × 3 kernel). The rectified linear unit (ReLU) function was used as an activation function for both convolution layers. The ReLU function returned the same value with positive input values, and returned zero with negative input values. The max-pooling layer, followed by the convolution layers, compressed the input data for operation reduction. A max-pooling layer followed another convolution layer (Conv3, 3 × 3 kernel) with the ReLU function. A flattened layer was then used to convert the data into a one-dimensional array. Subsequently, the fully connected dense layer in which each neuron in the dense layer was connected to all neurons of the previous layer were used. Finally, a fully connected dropout layer was used to avoid overfitting by randomly removing several neurons. The total number of input data were nine hundred seventy-five in the LIBS spectra. After randomly mixing the entire data, the data were divided into a training set and a test set at a ratio of 7:3. The CNN model was trained using the training set and verified using the test set. The CNN model was developed using Python, version 3.8 (Guido van Rossum, Delaware, USA).

## 3. Results and Discussion

### 3.1. Laser Cleaning

**Figure 2:**
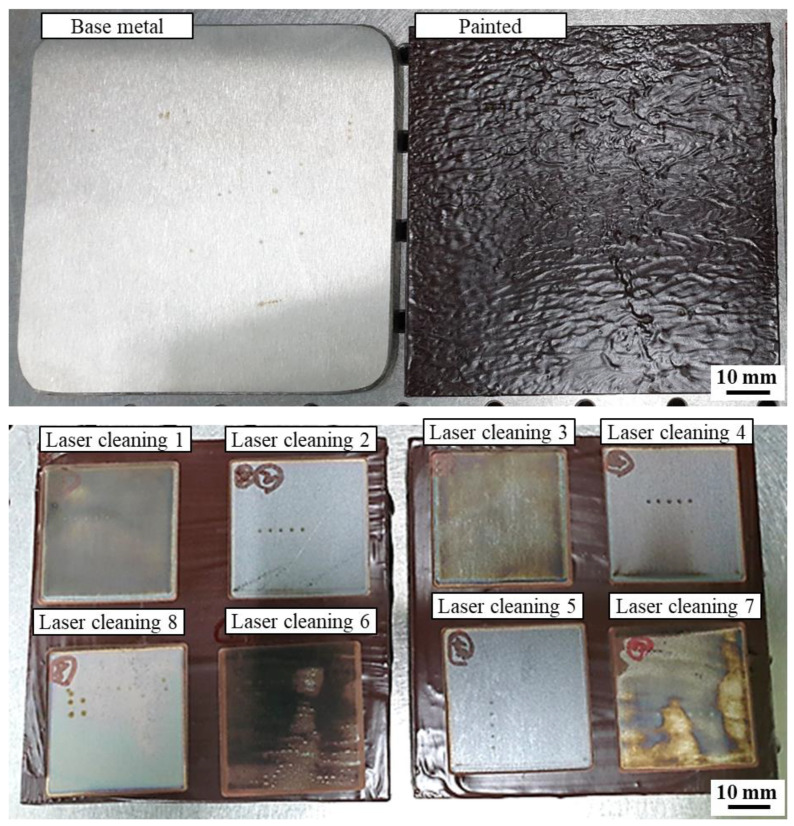
Images of the base metal, painted, and laser paint-cleaned specimens.

The laser paint removal level differed for the eight laser-cleaned specimens and strongly depended on the laser cleaning conditions (Figure 2). The paint was effectively removed for the LC2, LC4, LC5, and LC8, while the paint residue was still detected at the surface of LC1, LC3, LC6, and LC7. When the same laser power and scan speed were used, the paint removal level was improved with an increase in the number of repetitions. This was because the laser energy input increased as the repetition time increased. Moreover, when the laser power and scan speed differed, the paint was effectively removed with a higher laser energy input. However, the LC1 and LC3 showed poor paint removal level compared with the LC8, even though the laser energy input of the LC1 (0.5 J/mm) and LC3 (0.4 J/mm) was larger or same with that of the LC8 (0.4 J/mm). This was because the repetition of the laser cleaning process decreased the ablation threshold energy of the paint due to the incubation effect [18], inducing effective laser paint removal.

The EDS analysis was performed on the laser-cleaned specimens for quantitative analysis of the paint residue (Table 2). Low concentrations of C and high concentrations of O were detected for the LC2, LC4, LC5, and LC8 because of effective paint removal and oxide layer formation on the surface. In contrast, high concentrations of C and low concentrations of O were characterized for the LC1, LC3, LC6, and LC7, where the paint remained on the surface. In addition, the concentration of O tended to increase when the specimens were better cleaned owing to the generation of an oxide layer. It may be possible to evaluate the laser paint removal level by the EDS analysis; however, this is not appropriate for an in-line monitoring. 

### 3.2. LIBS Spectra

**Figure 3:**
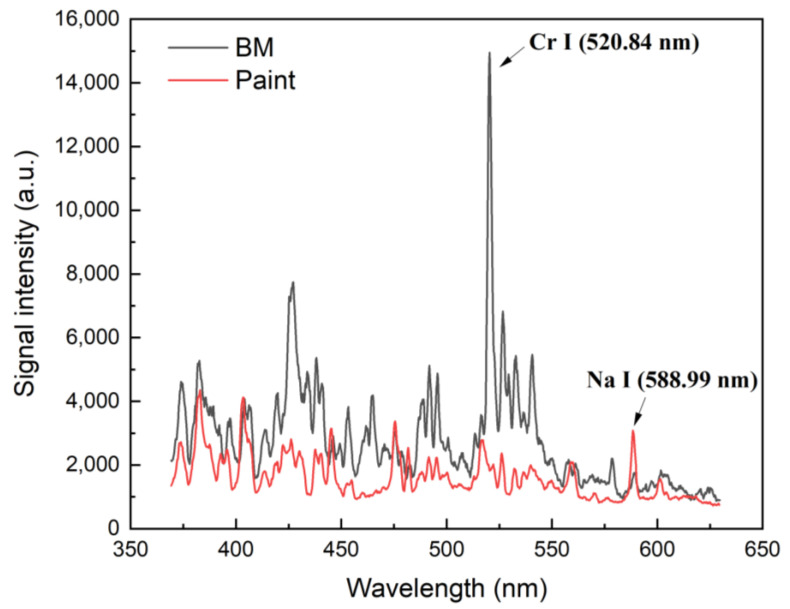
LIBS spectra of the base metal and painted specimen.

The LIBS analysis was employed to monitor the laser cleaning process. Figure 3 shows the LIBS spectra of the base metal (SS304L) and painted specimen. The major elements in SS304L were Fe, Cr, and Ni. In the spectral range of 370–630 nm, a strong Cr I (neutral atom) emission line appeared at 520.84 nm. For the painted specimen, a Na I emission line was detected with a distinguishable peak intensity at 588.99 nm compared to the base metal.

**Figure 4:**
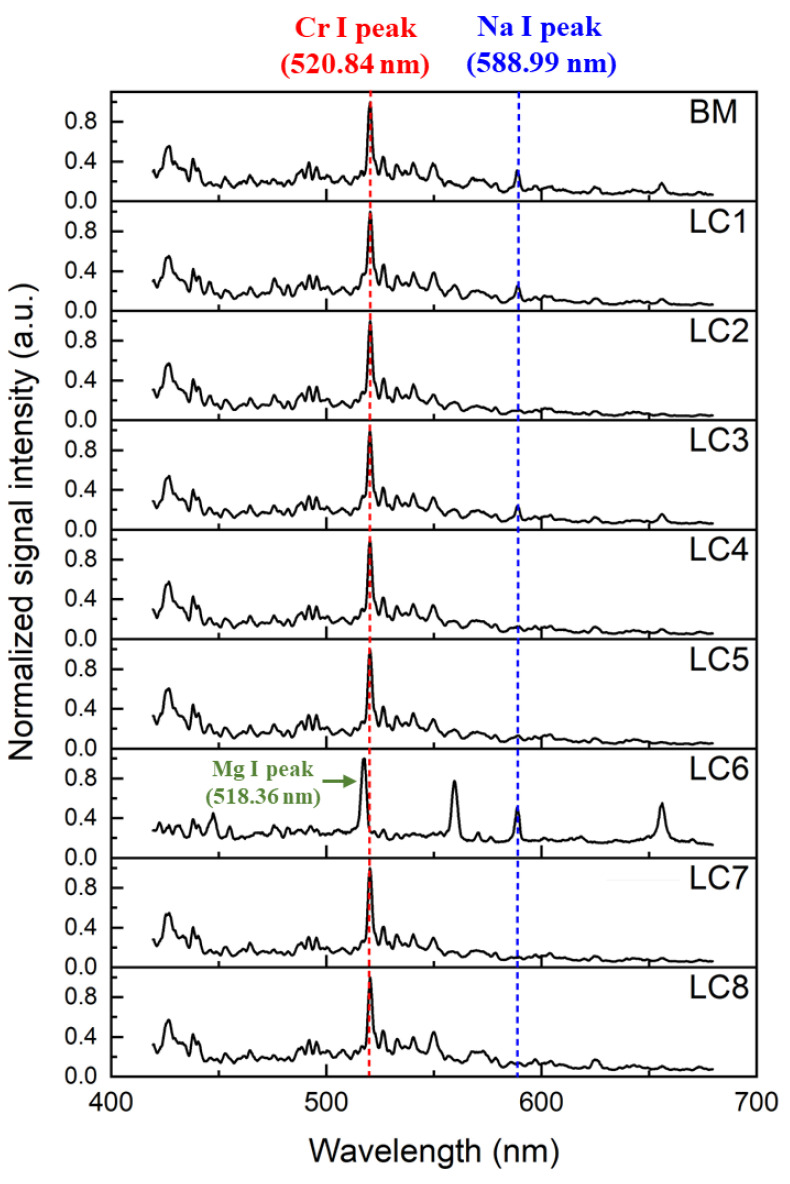
Normalized LIBS spectra of the base metal and laser-cleaned specimens.

Figure 4 shows the normalized LIBS spectra of the base metal and the LC specimens. The LC6 shows a different LIB spectrum compared to the other specimens owing to significant paint residue on the surface. A relatively strong Na emission line was detected at 588.99 nm for the LC6. Moreover, a strong Mg I peak was detected at 518.36 nm next to a Cr emission line for the LC6. Meanwhile, specimens other than the LC6 showed significantly similar LIBS spectra. In addition, a small concentration of Na was detected in the BM, which may have been contaminated during sample preparation (e.g., fingerprints). These factors make the classification of the LIBS spectrum more difficult. Therefore, the classification of the LIBS spectrum requires additional complex peak analyses, including background removal, peak intensity or peak area calculation, and peak intensity ratio calculation, for high accuracy and rapid analysis.

### 3.3. Classification Based on Convolution Neural Networks

**Figure 5:**
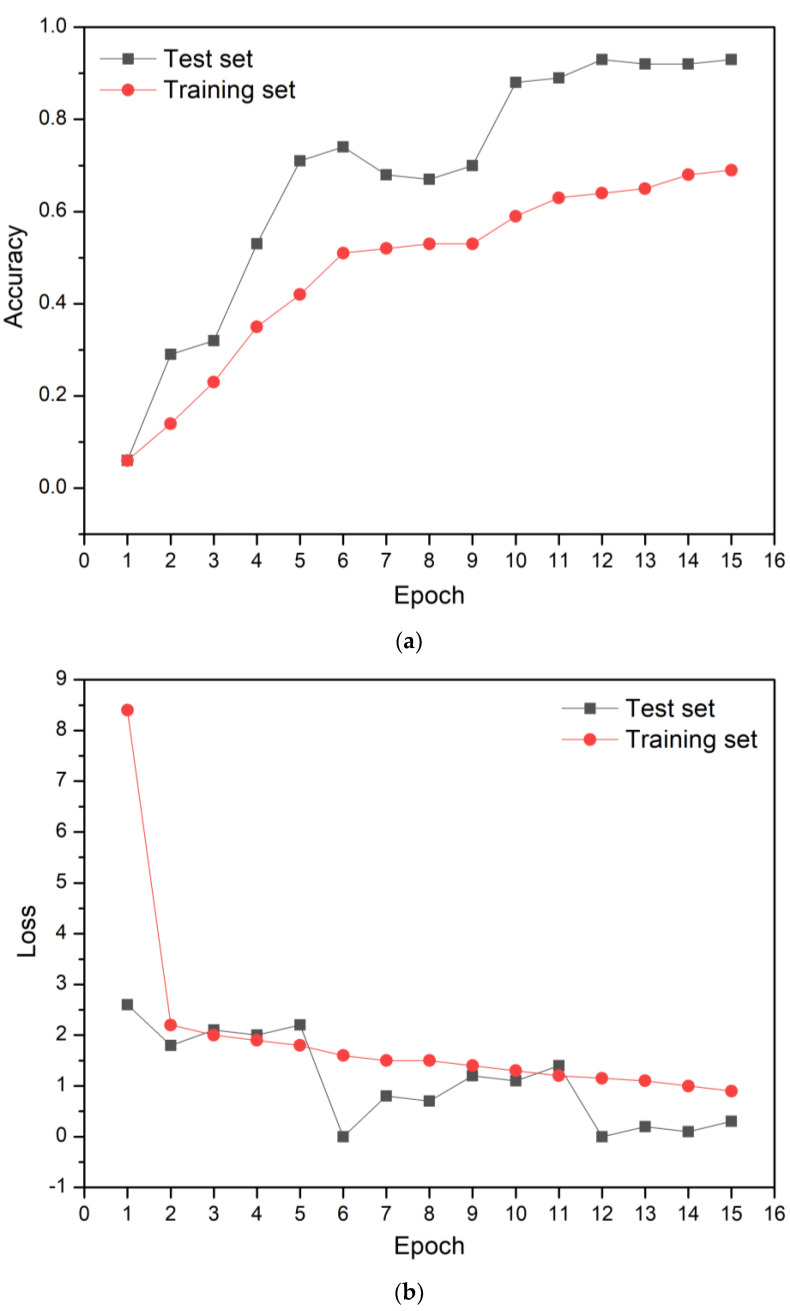
The learning (**a**) accuracy and (**b**) loss of the CNN model for 15 epochs.

**Figure 6:**
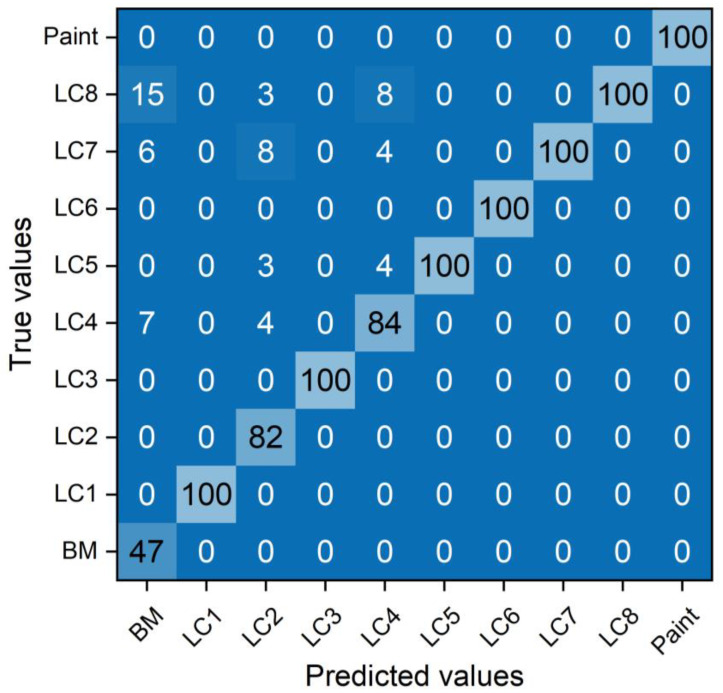
Confusion matrix after 15 epochs.

All spectral data were converted into image files by removing the layers, scales, grids, and labels. The developed CNN model was trained by repeating 15 and 20 learning times (i.e., epochs). Figure 5 shows the learning accuracy and loss of the CNN model after 15 epochs. Learning accuracy represents the number of prediction errors for the entire dataset. The loss is the difference between the true and predicted values (calculated using the CNN model). The learning accuracy and loss were 93% and 0.3 after 15 epochs. The performance of the CNN model can be evaluated with the accuracy of the test set. Therefore, the test set were used to evaluate the performance of the CNN model after the completion of training process. Figure 6 shows the confusion matrix after 15 epochs. If the true and predicted values are equivalent, it can be considered to have high learning accuracy. In other words, the CNN model can accurately classify BM, painted, and LC specimens. The painted, LC1, LC3, LC5, LC6, LC7, and LC8 showed 100% agreement between the true and predicted values.

In contrast, BM, LC2, and LC4 showed lower classification accuracy. For the LC4, it was classified as the LC4 itself eighty-four times. However, it was also predicted as the LC5 (four times), LC7 four times, and LC8 (eight times). These results indicate that the predicted values calculated by the CNN model (15 epochs) were not perfectly equivalent to the true values, resulting in a relatively low classification accuracy.

**Figure 7:**
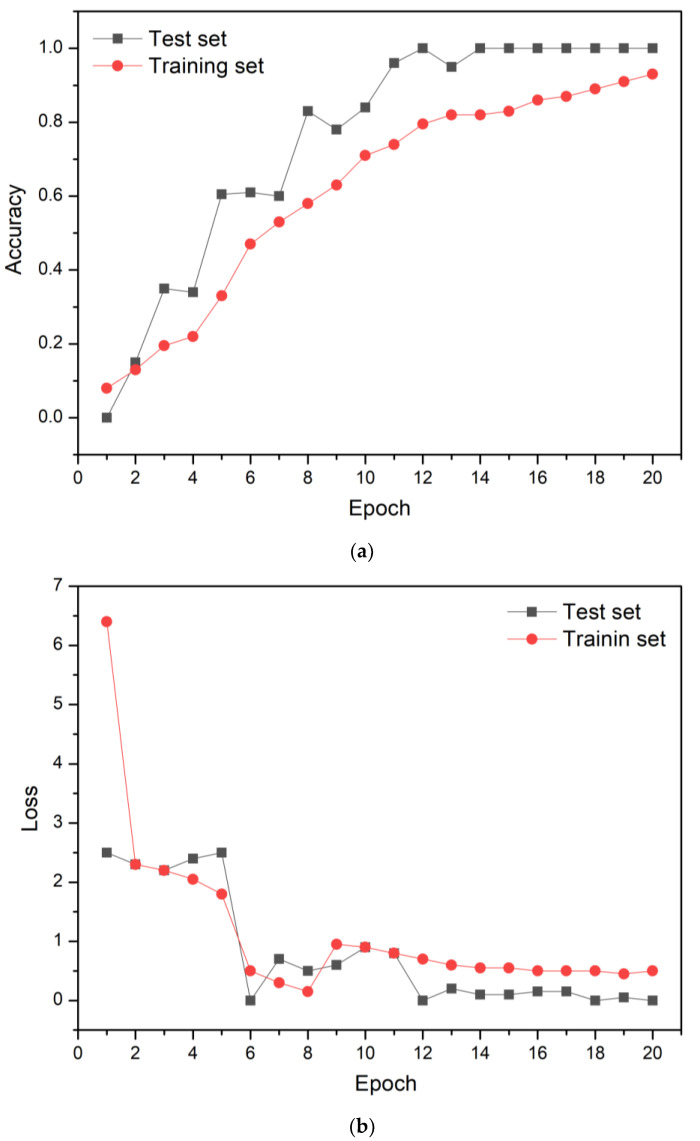
The learning (**a**) accuracy and (**b**) loss of the CNN model for 20 epochs.

**Figure 8:**
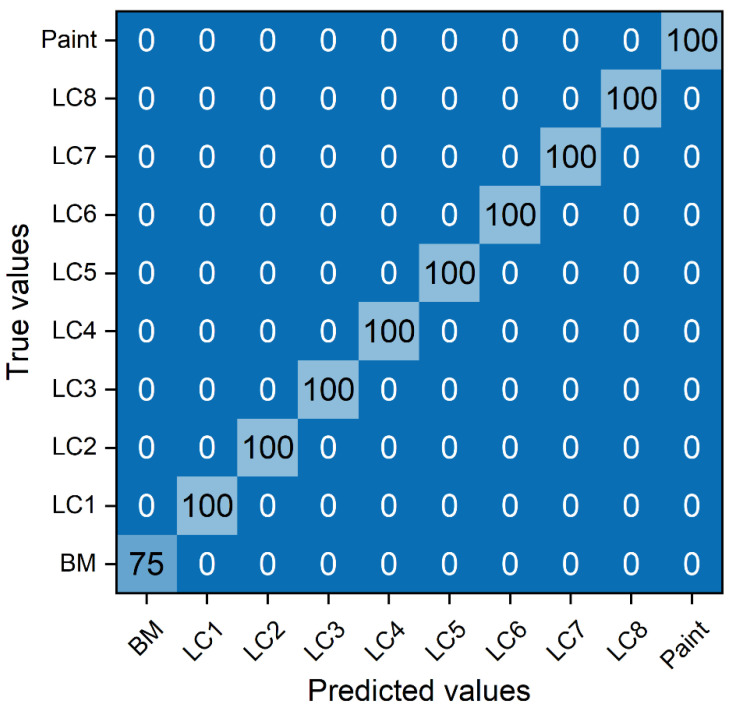
Confusion matrix after 20 epochs.

Figure 7 shows the learning accuracy and loss of the CNN model after 20 epochs. The learning accuracy improved to 100%, and the loss decreased to zero after 20 epochs. Figure 8 shows the confusion matrix after 20 epochs. The LIBS spectra analysis with the CNN model shows a perfect classification for every specimen (i.e., BM for seventy-five predictions and painted and LC specimens for one hundred predictions). In other words, the predicted values of the CNN model agreed perfectly with the true values without prediction errors. These results verified that excellent classification accuracy of the BM, painted, and LC specimens was achieved using the LIBS spectra analysis with the CNN model.

To verify the accuracy of the CNN model, the input data, which were not used for model training, were applied to the CNN model. The input data was classified as the most similar specimen among the ten outputs (i.e., BM, LC1~8, and paint) in Figure 1. The match rate represents the possibility of input data to be classified as the ten outputs. For example, the CNN model can classify the input data of LC2 as 20% of BM and 80% of LC2. In this case, the CNN model will classify the input data as the LC2 with a match rate of 80%. However, the CNN model will fail to classify the input data as the LC2 with a match rate of 90%. In other words, a high accuracy of the CNN model with the high match rates indicates the well-developed CNN model. The CNN model accuracy was 60% after 15 epochs, and it increased to 94.55% after 20 epochs for a match rate of 90% (Table 3). Moreover, the spectrum analysis time (measured by the time function in the Python program) only took 0.09 s. 

To predict the paint removal level by the CNN model, one hundred LIBS spectra of each laser-cleaned specimen were compared with that of the BM. Table 4 shows the number of LIBS spectra which showed the match rate of 90% with that of the BM. The well laser-cleaned specimens (i.e., LC2, LC4, LC5, and LC8) showed relatively higher count value, while the poor laser-cleaned specimens with the presence of paint residue on the surface (i.e., LC1, LC3, LC6, and LC7) achieved relatively lower count value (lower than eighty). In particular, the LC6, the poorest laser-cleaned specimen, showed zero count value. This result verified that the LIBS-coupled CNN method can determine the laser paint removal level.

Table 3 and Table 4 show that the LIBS spectra for the laser cleaning process were analyzed with high accuracy and rapid measurements using the CNN model. Therefore, the LIBS spectra analysis with the CNN model can be adopted for an in-line monitoring of the laser cleaning process.

## 4. Conclusions

In this study, painted SS304L specimens were laser-cleaned using different process parameters such as laser power, scan speed, and the number of repetitions. Eight different specimens were produced with different paint removal levels. The LIBS spectra were acquired at the surface of the laser-cleaned specimens to evaluate the paint residue. The CNN-based deep learning model was developed to accurately and rapidly identify laser-cleaned specimens. The learning accuracy was 100% after 20 epochs. The CNN model accuracy of 94.55% was achieved after 20 epochs when the match rate between the input data and learning data was 90%. The analysis time of a spectrum only took 0.09 s. The LIBS-coupled CNN method successfully distinguished eight different LIBS spectra and determined the paint removal level. This is the first study to verify the feasibility of LIBS spectra analysis using the CNN model as an in-line monitoring technique for the laser cleaning process.

## Figures and Tables

**Table 1:** Process parameters of laser cleaning.

	LC1	LC2	LC3	LC4	LC5	LC6	LC7	LC8
Power (W)	500	500	400	400	400	400	400	400
Scan speed (mm/s)	1000	1000	1000	1000	1000	3000	3000	3000
Number of repetitions	1	2	1	2	3	1	2	3
Laser energy input (J/mm)	0.5	1	0.4	0.8	1.2	0.13	0.27	0.4

**Table 2:** Results of EDS analysis (wt%).

Element	LC1	LC2	LC3	LC4	LC5	LC6	LC7	LC8
C	25.47	2.73	15.35	5.42	2.97	49.42	11.69	2.81
O	17.02	31.32	6.41	20.45	30.77	11.52	4.12	30.28
Cr	3.39	10.72	13.53	12.27	12.91	4.86	15.93	10.84
Mn	7.92	4.67	2.47	3.82	3.00	6.77	0.85	4.81
Fe	45.66	50.42	57.17	54.81	49.19	27.18	60.67	50.59
Ni	0.54	0.14	5.07	3.24	1.15	0.25	6.73	0.67
Total:	100.00	100.00	100.00	100.00	100.00	100.00	100.00	100.00

**Table 3:** The accuracy of the CNN model.

15 Epochs		20 Epochs	
Match rate with Learning Data (%)	Accuracy of Output (%)	Match Rate with Learning Data (%)	Accuracy of Output (%)
95	47.27	95	89.09
90	60.00	90	94.55
80	69.09	80	100.00
70	87.27	70	100.00
60	89.09	60	100.00

**Table 4:** The number of almost laser cleaned spectra.

	LC1	LC2	LC3	LC4	LC5	LC6	LC7	LC8
Count (ea)	77	89	73	88	92	0	74	96

## Data Availability

The raw and processed data required to reproduce these findings are available upon request from the corresponding author.
